# Micrometer Backstepping Control System for Linear Motion Single Axis Robot Machine Drive

**DOI:** 10.3390/s19163616

**Published:** 2019-08-20

**Authors:** Chih-Hong Lin, Kuo-Tsai Chang

**Affiliations:** Department of Electrical Engineering, National United University, 36063 Miaoli, Taiwan

**Keywords:** ant colony optimization, backstepping control, Gottlieb polynomials neural network, Lyapunov function, linear motion single axis robot machine

## Abstract

In order to cut down influence on the uncertainty disturbances of a linear motion single axis robot machine, such as the external load force, the cogging force, the column friction force, the Stribeck force, and the parameters variations, the micrometer backstepping control system, using an amended recurrent Gottlieb polynomials neural network and altered ant colony optimization (AACO) with the compensated controller, is put forward for a linear motion single axis robot machine drive system mounted on the linear-optical ruler with 1 um resolution. To achieve high-precision control performance, an adaptive law of the amended recurrent Gottlieb polynomials neural network based on the Lyapunov function is proposed to estimate the lumped uncertainty. Besides this, a novel error-estimated law of the compensated controller is also proposed to compensate for the estimated error between the lumped uncertainty and the amended recurrent Gottlieb polynomials neural network with the adaptive law. Meanwhile, the AACO is used to regulate two variable learning rates in the weights of the amended recurrent Gottlieb polynomials neural network to speed up the convergent speed. The main contributions of this paper are: (1) The digital signal processor (DSP)-based current-regulation pulse width modulation (PWM) control scheme being successfully applied to control the linear motion single axis robot machine drive system; (2) the micrometer backstepping control system using an amended recurrent Gottlieb polynomials neural network with the compensated controller being successfully derived according to the Lyapunov function to diminish the lumped uncertainty effect; (3) achieving high-precision control performance, where an adaptive law of the amended recurrent Gottlieb polynomials neural network based on the Lyapunov function is successfully applied to estimate the lumped uncertainty; (4) a novel error-estimated law of the compensated controller being successfully used to compensate for the estimated error; and (5) the AACO being successfully used to regulate two variable learning rates in the weights of the amended recurrent Gottlieb polynomials neural network to speed up the convergent speed. Finally, the effectiveness of the proposed control scheme is also verified by the experimental results.

## 1. Introduction

A linear motion single axis robot machine that can achieve rapid rates of acceleration by use of electromagnetic force has few features which are of merit [1,2,3], such as being simple fabric, having no adverse reaction, little friction, elated velocity, elated pushed force, and elated precision in a long-distance location and so on. A linear motion single axis robot machine consists of some of magnets that create constant magnetic fields, and some windings that create the traveling magnetic fields. A number of applications of the linear motion single axis robot machine include checking the camera moving unit, ink jet printer, chip mounter, checking the device, a high-speed screw-tightening unit, a high-speed loading/unloading robot, and material handling systems [1,2,3]. 

One of the control methods for the large state feedback linearizable systems include the backstepping techniques [3,4,5]. The design of tracking and adjustment strategies can provide a systematic skeleton. Moreover, to extend to the estimation of unknown parameters of the system, the adaptive backstepping methods [6,7] were put forward to estimate some unknown parameters of the system. Furthermore, some adaptive backstepping controllers were used for some linear machines [8,9] to estimate uncertainty. In addition, some neural networks [10,11,12] have been used for the nonlinear systems to estimate unknown parameters for uncertainty. Therefore, the adaptive backstepping controllers, combined with some neural networks [13,14,15] are generally applied to control the nonlinear systems so as to estimate some uncertainties and enhance system robustness. However, these methods are only limited to the bounded region, and have never showed any compensated mechanics or technology. Thus, the motivation of the proposed micrometer backstepping control system, by means of the amended recurrent Gottlieb polynomials neural network and AACO with the compensated controller for a linear motion single axis robot machine mounted with a linear optical-ruler sensor with 1 um precision and three Hall sensors, provides an estimated method and error compensation mechanism which can be used to enhance the robustness of the system under parameter variations and external force disturbances to raise the control precision. 

Due to lesser computational complexity and faster convergent speed, the polynomials-function neural networks [16,17] have recently been proposed to reduce computational costs, while some parts of the polynomials-function neural networks were used to estimate some unknown parameters or the lumped uncertainty. Owing to uncertain actions, the control performance of the linear motion single axis robot machine drive can have a serious influence. The micrometer backstepping control system using an amended recurrent Gottlieb polynomials neural network [18,19,20] and altered ant colony optimization (AACO) [21,22] with the compensated controller has thus been put forward to control the motion position of the linear motion single axis robot machine to track periodic references.

This paper presents the micrometer backstepping control system using an amended recurrent Gottlieb polynomials neural network and AACO with the compensated controller, which has an error estimated law with an adaptive law, to control the linear motion single axis robot machine drive system so as to enhance the robustness of the system under the parameter variations and the external load force disturbances. The amended recurrent Gottlieb polynomials neural network with an adaptive law is too proposed to adapt the value of the lumped uncertainty. Besides, the compensated controller with an adaptive law by use of the novel error estimated law is also proposed to compensate for the estimated error between the lumped uncertainty and the amended recurrent Gottlieb polynomials neural network. Moreover, the AACO is used to regulate two variable learning rates in the weights of the amended recurrent Gottlieb polynomials neural network to speed up the convergent speed. The important contributions of this paper are: (1) The digital signal processor (DSP)-based current-regulation pulse width modulation (PWM) control scheme being successfully applied to control the linear motion single axis robot machine drive system; (2) the micrometer backstepping control system using an amended recurrent Gottlieb polynomials neural network with the compensated controller being successfully derived according to the Lyapunov function to diminish the lumped uncertainty effect; (3) achieving high-precision control performance, where an adaptive law of the amended recurrent Gottlieb polynomials neural network based on the Lyapunov function is successfully applied to estimate the lumped uncertainty; (4) a novel error-estimated law of the compensated controller being successfully used to compensate for the estimated error; and (5) the AACO being successfully used to regulate two variable learning rates in the weights of the amended recurrent Gottlieb polynomials neural network to speed up the convergent speed. Finally, the effectiveness of the proposed control scheme is also verified by the experimental results.

## 2. Materials and Methods 

### 2.1. Model of Linear Motion Single Axis Robot Machine

The *d*-*q* axis model of the linear motion single axis robot machine by use of a synchronous rotating reference frame can be described as follows [3]:

(1) $$v_{qs}=R_{1s}i_{qs}+L_{qs}di_{qs}/dt+\omega_{es}(L_{ds}i_{ds}+\lambda_{pms})$$

(2) $$v_{ds}=R_{1s}i_{ds}+L_{ds}di_{ds}/dt-\omega_{es}L_{qs}i_{qs}$$

where $v_{ds}\;,\;v_{qs}$ are the *d-*axis and *q-*axis voltages; $i_{ds}\;,\;i_{qs}$ are the *d*-axis and *q-*axis currents; $R_{1s}$ is the phase winding resistance; $L_{ds}\;,\;L_{qs}$ are the *d-*axis and *q-*axis inductances; $\omega_{es}=P_{s}\;\omega_{rs}$ is the electrical angular velocity; $\omega_{rs}$ is the angular velocity of the mover; and $\lambda_{pms}$ is the permanent magnet flux linkage. Besides,

(3) $$\omega_{rs}=\pi \;v_{rs}/\delta$$

(4) $$v_{e}=P_{s}\;v_{rs}=2\delta \;f_{es}$$

where $P_{s}$ is the number of pole pairs; $v_{r}$ is the linear velocity; δ is the pole pitch; $v_{e}$ is the electrical linear velocity; and $f_{es}$ is the electrical frequency. The electromagnet-pushed force [3] is given by

(5) $$F_{e}=3\pi P_{s}[\lambda_{pms}i_{qs}+(L_{ds}-L_{qs})\;i_{ds}i_{qs}]/2\delta$$

Then, the electromagnetic-pushed power [1,2,3] is given by

(6) $$P_{e}=F_{e}v_{e}=3P_{s}[\lambda_{pms}i_{qs}+(L_{ds}-L_{qs})\;i_{ds}i_{qs}]\omega_{es}/2$$

and the dynamic equation of the mover in the linear motion single axis robot machine drive system is given by

(7) $$M_{s}dv_{r}/dt+D_{s}v_{r}=F_{e}-F_{l}-F_{r}-F_{f}-F_{c}$$

where $F_{e}$ is the electromagnet-pushed force; $M_{s}$ is the total mass of the moving element system; $D_{s}$ is the viscous friction; $F_{l}$ is the external load-pushed force which satisfies the condition $\left|F_{l}\right|\leq m_{p}$; $F_{r}=k_{a}sign(v_{r})$ is the Stribeck effect force; $F_{f}=k_{b}sign(v_{r})$ is the coulomb friction force; and $F_{c}$ is the cogging force from the slotting and the finite length of the iron-cored stator.

### 2.2. Drive System of Linear Motion Single Axis Robot Machine

**Figure 1:**
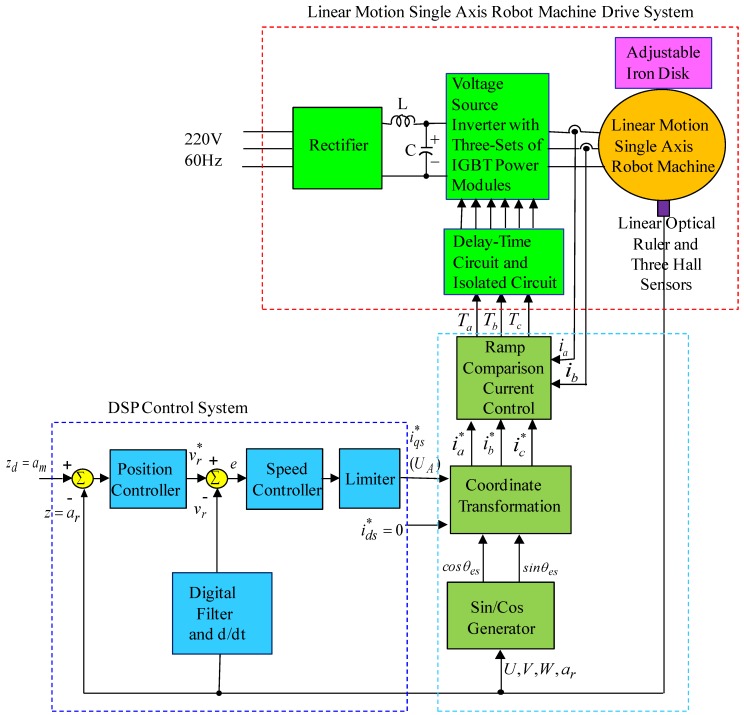
Makeup of linear motion single axis robot machine and drive system.

The linear motion single axis robot machine is made up of a linear motor and a linear slider. A linear motor-driven linear slider module is equipped with a stainless-steel cover strip to prevent particles from entering or exiting. The basic control approach of the linear motion single axis robot machine drive system adopted the field orientation [1,2,3]. For the field-oriented control, the rotor flux is produced by the *d-*axis only, while the current vector is generated by the *q-*axis. When $i_{ds}$ is equal to zero and the flux linkage $\lambda_{pms}$ is a fixed value, then the electromagnet-pushed force $F_{e}$ is proportional to $i_{qs}^{*}$ from (5) and (6). The electromagnetic force is linearly proportional to the *q-*axis current when the *d-*axis flux is constant in (5), where the maximum force per ampere can be achieved. The electromagnet-pushed force equation from (5) can be rewritten by [3],

(8) $$F_{e}=3\pi P_{s}\lambda_{pms}i_{qs}/(2\delta )=K_{f}i_{qs}^{*}$$

where $K_{f}=3\pi \;P_{s}\lambda_{pm}/(2\delta )$ is the electromagnetic-pushed force coefficient, and $i_{qs}^{*}$ is the command of the electromagnetic-pushed force current. The makeup of a field-oriented linear motion single axis robot machine drive system is shown in Figure 1. The linear motion single axis robot machine drive system incorporates a linear motion single axis robot machine, a ramp comparison current-controlled pulse width modulation (PWM) voltage source inverter (VSI), a field-orientation mechanism, a coordinate translator, a speed control loop, a position control loop, a linear-optical ruler, and three Hall sensors [3]. The detection of the motion position was used by a linear-optical ruler with 1 um resolution. The detection of the permanent magnet (PM) position was used by three Hall sensors with three signals as *U*, *V,* and *W*. The output signals of three Hall sensors, which consist of the Hall elements and the associated electronics elements, are the rectangular analog signals [3]. Some different sizes of iron disks were used to change the mass of the mover and viscous friction of the motion mover of the linear motion single axis robot machine. 

**Figure 2:**
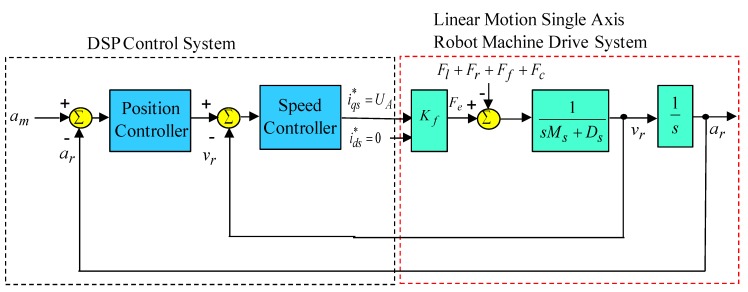
Simplified block diagram of linear motion single axis robot machine drive system.

The digital signal processor (DSP) control system by TMS32C32 chip was used to execute the field-oriented control. With the implementation of field-oriented control [1,2,3], the simplified block diagram of the linear motion single axis robot machine drive system is shown in Figure 2. The specifications of the linear motion single axis robot machine are given as 220 V, 3.1 A, 0.6 kW, 50.8 N, with 0.1 m distance, 0.02 m width. For the convenience of the controller design, the speed and position signals were set at 1 V = 200 µm/s and 1 V = 200 µm. The electrical parameters of the linear motor of the linear motion single axis robot machine are given as $M_{s}=\;2.1\;\mathrm{kg}=0.1812\;N\cdot s/V$, $D_{s}=81.62\;\mathrm{kg}/s=5.021\;N/V$, $K_{f}=32.2\;N/A$.

### 2.3. Micrometer Backstepping Control System Using an Amended Recurrent Gottlieb Polynomials Neural Network and AACO with the Compensated Controller

By using (7) and (8), the dynamic equation for the linear motion single axis robot machine drive, including the external load force, the cogging force, the column friction force, the Stribeck effect force, and the parameters’ variations can be represented as:

(9) $${\dot{x}}_{r}=h_{r}x_{r}+l_{r}U_{A}+\Delta h_{r}x_{r}+\Delta l_{r}U_{A}+n_{r}(F_{l}+F_{r}+F_{f}+F_{c})=h_{r}x_{r}+l_{r}U_{A}+f_{u}$$

where $a_{r}$ is the motion position of the linear motion single axis robot machine, ${\dot{a}}_{r}=v_{r}=x_{r}$ is the motion velocity of the linear motion single axis robot machine, and $h_{r}=-D_{s}/M_{s}$, $l_{r}=K_{f}/M_{s}>0$ and $n_{r}=-1/M_{s}$ are three real numbers. $f_{u}=\Delta h_{r}x_{r}+\Delta l_{r}U_{A}+n_{r}(F_{l}+F_{r}+F_{f}+F_{c})$ is the lumped uncertainty that includes the external load force, the cogging force, the column friction force, the Stribeck effect force, and the parameters’ variations. $U_{A}=i_{qs}^{*}$ is the control intensity of the linear motion single axis robot machine drive system—that is, the command of electromagnetic-pushed force current.

The tracking error of the motion position is defined by:

(10) $$q_{1}=a_{m}-a_{r}=z_{d}-z$$

Differential (10) is:

(11) $${\dot{q}}_{1}={\dot{z}}_{d}-\dot{z}={\dot{a}}_{m}-{\dot{a}}_{r}={\dot{z}}_{d}-x_{r}$$

The stabilizing function is defined by:

(12) $$\gamma_{1}=m_{1}q_{1}+{\dot{z}}_{d}+m_{2}\nu$$

where $m_{1}$ and $m_{2}$ are two real numbers greater than zero, and $\nu =\int q_{1}(\tau )d\tau$ is the integral function [3]. The virtual tracking error of motion position is defined by

(13) $$q_{2}=x_{r}-\gamma_{1}$$

By use of (9) and (13), the differential of (13) is given by

(14) $${\dot{q}}_{2}={\dot{x}}_{r}-{\dot{\gamma }}_{1}=(h_{r}x_{r}+l_{r}U_{A}+f_{u})-{\dot{\gamma }}_{1}=h_{r}(q_{2}+\gamma_{1})+l_{r}U_{A}+f_{u}-{\dot{\gamma }}_{1}$$

**Figure 3:**
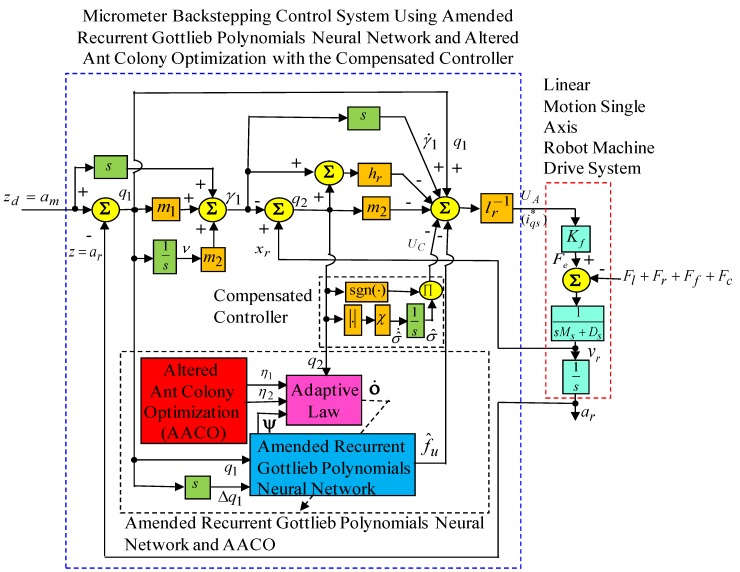
Micrometer backstepping control system using an amended recurrent Gottlieb polynomials neural network and altered ant colony optimization with the compensated controller.

The control objective is to track the reference trajectory $a_{m}=z_{d}(t)$ asymptotically. In advance for practical applications, the lumped uncertainty $f_{u}$ is difficult to know. Because the lumped uncertainty $f_{u}$ is difficult to measure in practical applications, and the upper bound ${\bar{f}}_{u}>f_{u}$ is difficult to know, an amended recurrent Gottlieb polynomials neural network uncertainty observer has been proposed to adapt the value of the lumped uncertainty,$f_{u}$. In consequence, the micrometer backstepping control system using an amended recurrent Gottlieb polynomials neural network and AACO with the compensated controller, which is shown in Figure 3, is proposed for tracking of the reference trajectory $a_{m}=z_{d}(t)$ under the lumped uncertainty $f_{u}$, assuming that $z_{d}(t)$, ${\dot{z}}_{d}(t)$, and ${\ddot{z}}_{d}(t)$ are all bounded functions. Additionally, the estimation of the rehabilitated error Q is compensated for by the controller with an adaptive law to compensate for the observed error that is based on the Lyapunov function to further guarantee the stable characteristic of the whole control system. Furthermore, in order to train the amended recurrent Gottlieb polynomials neural network effectively, an online parameter training methodology and the updated law was derived by means of the Lyapunov stability theorem and the gradient descent method. In order to raise convergent speed, the AACO was used to regulate two variable learning rates in the weights of the amended recurrent Gottlieb polynomials neural network. 

**Figure 4:**
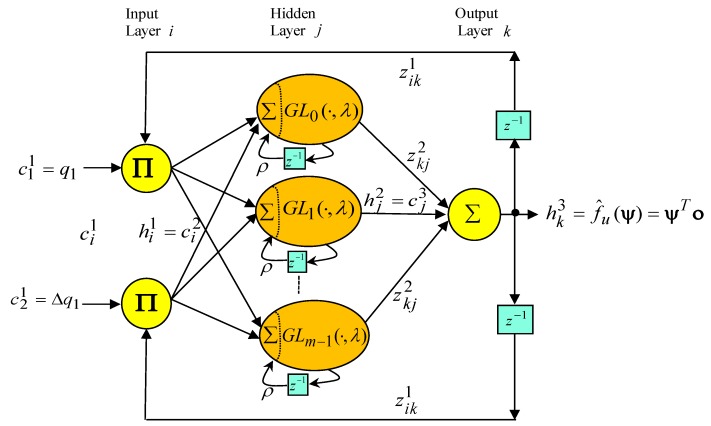
Makeup of the three-layer amended recurrent Gottlieb polynomials neural network.

The makeup of the proposed three-layer amended recurrent Gottlieb polynomials neural network, which is made up of the input layer, the hidden layer, and the output layer, is shown in Figure 4. 

All signal actions in every node of the three layers can be described as follows:

(15) $$dn_{i}^{1}(N)=\prod_{k}h_{i}^{1}(N)z_{ik}^{1}\;h_{k}^{3}(N-1),h_{i}^{1}(N)=dn_{i}^{1}(N),\;i=1,\;2$$

(16) $$dn_{j}^{\text{​​ }2}(N)=\sum_{i=1}^{2}h_{i}^{1}(N)+\rho \;h_{j}^{2}(N-1),\;h_{j}^{2}(N)=GL_{j}(dn_{j}^{2}(N),\lambda ),\;j=0,\;1,\;2,\cdots ,(m-1)$$

(17) $$dn_{k}^{\;3}(N)=\sum_{j=0}^{m-1}z_{kj}^{2}\;h_{j}^{2}(N),h_{k}^{3}(N)=dn_{k}^{3}(N),\;k=1$$

where *N* denotes the number of iterations. ∏ and Σ are the multiplication operator and the summation operator, respectively. $c_{1}^{1}=q_{1}$ and $c_{2}^{1}=q_{1}(1-z^{-1})=\Delta q_{1}$ are the tracking error and the tracking error increment, respectively. $z_{ik}^{1}$ and $z_{kj}^{2}$ are the recurrent weight from the output layer to the input layer and the connective weight from the hidden layer to the output layer, respectively. $h_{i}^{1}$, $h_{j}^{2}$ , and $h_{k}^{3}$ are the output value from the input layer, the output value from the hidden layer, and the output value from the output layer, respectively. ρ is the self-feedback gain of the hidden layer. For the Gottlieb polynomials [18,19,20], $GL_{n}(x,\lambda )$ is the argument of the polynomials with −1<x<1, and *n* is the order of expansion. The zero-, first-, and second-order Gottlieb polynomials are given by $GL_{0}(x,\lambda )=1$ , $GL_{1}(x,\lambda )=-0.5e^{-2\lambda }(-1-x+xe^{\lambda })$, and $GL_{2}(x,\lambda )=-0.5e^{-2\lambda }(-2-3x+2xe^{\lambda }-x^{2}+2x^{2}e^{\lambda }-e^{2\lambda x^{2}}+e^{2\lambda x})$, respectively. The higher-order Gottlieb polynomials may be generated by Gottlieb [18,19,20]. Two activation functions $h_{i}^{1}$ and $h_{k}^{3}$ were adopted in the linear functions. 

The output value $h_{k}^{3}(N)$ of the amended recurrent Gottlieb polynomials neural network can be denoted by:

(18) $$h_{k}^{3}(N)={\hat{f}}_{u}(\psi )=\psi^{T}ο$$

where $\psi ={\left[z_{10}^{2}\;z_{11}^{2}\;\cdots \cdots \;z_{1,m-1}^{2}\right]}^{\text{​ }T}$ is the collections of the adjustable parameters of the amended recurrent Gottlieb polynomials neural network, and $c_{j}^{3}(N)=h_{j}^{2}(N)$ represents the *j*th input to the node of the output layer, and $ο={\left[c_{0}^{3}\;c_{1}^{3}\;\cdots \cdots \;c_{m-1}^{3}\right]}^{T}$, in which $h_{j}^{2}$ is determined by the selected Gottlieb polynomials and $-1\leq h_{j}^{2}\leq 1$.

The minimum rehabilitated error is defined by:

(19) $$Q=f_{u}-{\hat{f}}_{u}(\psi^{*})=f_{u}-{\left(\psi^{*}\right)}^{T}ο$$

where Q is the minimum rehabilitated error, and the absolute value of Q is less than a small positive constant σ. That is, |Q|≤σ; $\psi^{*}$ is the best weight vector that can achieve the minimum rehabilitated error. To develop the adaptive law of the amended recurrent Gottlieb polynomials neural network and error-estimated law, the Lyapunov function is given by

(20) $$g_{b}=0.5q_{1}^{2}+0.5q_{2}^{2}+0.5m_{2}\nu^{2}+0.5{\tilde{\sigma }}^{2}/\chi +0.5{\left(\psi -\psi^{*}\right)}^{T}(\psi -\psi^{*})/\eta_{1}$$

where χ and $\eta_{1}$ are positive real numbers. Define the estimated error by

(21) $$\tilde{\sigma }=\hat{\sigma }-\sigma$$

where $\hat{\sigma }$ is the estimation value of σ. By use of (11), (12), (13), (14), and $\nu =\int q_{1}(\tau )d\tau$, the derivative of the (20) can be written by

(22) $${\dot{g}}_{b}=q_{1}{\dot{q}}_{1}+q_{2}{\dot{q}}_{2}+m_{2}\nu \dot{\nu }+\frac{\tilde{\sigma }\dot{\tilde{\sigma }}}{\chi }+\frac{{\left(\psi -\psi^{*}\right)}^{T}\dot{\psi }}{\eta_{1}}\;=q_{1}(-\gamma_{1}-m_{1}q_{1}-m_{2}\nu -q_{2}+\gamma_{1})+q_{2}[(h_{r}(q_{2}+\alpha_{1})+l_{r}U_{A}+f_{u})-{\dot{\gamma }}_{1}]+m_{2}\nu \dot{\nu }+\frac{\tilde{\sigma }\dot{\tilde{\sigma }}}{\chi }+\frac{{\left(\psi -\psi^{*}\right)}^{T}\dot{\psi }}{\eta_{1}}\;=q_{1}(-m_{1}q_{1}-q_{2})+q_{2}[(h_{r}(q_{2}+\gamma_{1})+l_{r}U_{A}+f_{u})-{\dot{\gamma }}_{1}]+\frac{\tilde{\sigma }\dot{\tilde{\sigma }}}{\chi }+\frac{{\left(\psi -\psi^{*}\right)}^{T}\dot{\psi }}{\eta_{1}}$$

Then, from (22), the control strength $U_{A}$ of the micrometer backstepping control using the amended recurrent Gottlieb polynomials neural network and AACO with the compensated controller is designed by

(23) $$U_{A}=l_{r}^{-1}[q_{1}-m_{2}q_{2}-h_{r}(q_{2}+\gamma_{1})-{\hat{f}}_{u}(\psi )-U_{c}+{\dot{\gamma }}_{1}]$$

Substituting (23) into (22), the following equation can be obtained by

(24) $${\dot{g}}_{b}=-m_{1}q_{1}{}^{2}-m_{2}q_{2}^{2}+q_{2}(f_{u}-{\hat{f}}_{u}(\psi )-U_{c})+\frac{\tilde{\sigma }\dot{\tilde{\sigma }}}{\chi }+\frac{{\left(\psi -\psi^{*}\right)}^{T}\dot{\psi }}{\eta_{1}}\;=-m_{1}q_{1}{}^{2}-m_{2}q_{2}^{2}+q_{2}(f_{u}-{\hat{f}}_{u}(\psi^{*}))-q_{2}({\hat{f}}_{u}(\psi )-{\hat{f}}_{u}(\psi^{*}))-q_{2}U_{c}+\frac{\tilde{\sigma }\dot{\tilde{\sigma }}}{\chi }+\frac{{\left(\psi -\psi^{*}\right)}^{T}\dot{\psi }}{\eta_{1}}\;=-m_{1}q_{1}{}^{2}-m_{2}q_{2}^{2}+q_{2}Q-q_{2}{\left(\psi -\psi^{*}\right)}^{T}ο-q_{2}U_{c}+\frac{\tilde{\sigma }\dot{\tilde{\sigma }}}{\chi }+\frac{{\left(\psi -\psi^{*}\right)}^{T}\dot{\psi }}{\eta_{1}}$$

In order to make ${\dot{g}}_{b}\leq 0$, the adaptive law for $\dot{\psi }$, the compensated controller $U_{c}$ with error-estimated laws, and the adaptive law of the estimated error $\dot{\hat{\sigma }}$ are designed as:

(25) $$\dot{\psi }=\eta_{1}\;q_{2}\;ο$$

(26) $$U_{c}=\hat{\sigma }\mathrm{sgn}(q_{2})$$

(27) $$\dot{\hat{\sigma }}=\dot{\tilde{\sigma }}=\chi \left|q_{2}\right|$$

Substitute (21), (25), (26), and (27) into (24). Then, (24) can be rewritten by

(28) $$\begin{array}{l} {\dot{g}}_{b}=-m_{1}q_{1}{}^{2}-m_{2}q_{2}^{2}+q_{2}Q-q_{2}{\left(\psi -\psi^{*}\right)}^{T}ο-q_{2}\hat{\sigma }\mathrm{sgn}(q_{2})+\frac{(\hat{\sigma }-\sigma )\chi \left|q_{2}\right|}{\chi }+\frac{{\left(\psi -\psi^{*}\right)}^{T}\eta_{1}\;q_{2}\;ο}{\eta_{1}}\\ =-m_{1}q_{1}{}^{2}-m_{2}q_{2}^{2}+q_{2}Q-\left|q_{2}\right|\hat{\sigma }+(\hat{\sigma }-\sigma )\left|q_{2}\right|\\ =-m_{1}q_{1}{}^{2}-m_{2}q_{2}^{2}+q_{2}Q-\sigma \left|q_{2}\right|\\ \leq -m_{1}q_{1}{}^{2}-m_{2}q_{2}^{2}+\left|q_{2}\right|(\left|Q\right|-\sigma )\\ \leq -m_{1}q_{1}{}^{2}-m_{2}q_{2}^{2}\\ \leq 0 \end{array}$$

Define the following term:

(29) $$\tau (t)=m_{1}q_{1}^{2}+m_{2}\;q_{2}^{2}\leq -\;{\dot{h}}_{2}$$

Then,

(30) $$\int_{\;0}^{\text{ ​}t}\tau (\tau )\;d\tau \leq h_{2}(q_{1}(0),\;q_{2}(0))-h_{2}(q_{1}(t),q_{2}(t))$$

Because $h_{1}(q_{1}(t),\;q_{2}(t))$ is nonincreasing and bounded, and $h_{1}(q_{1}(0),\;q_{2}(0))$ is bounded, then $\dot{\tau }\left(t\right)$ is bounded and τ(t) is uniformly continuous [23,24]; thus, $\underset{t\to \infty }{\lim}\tau (t)=0$ and $\underset{t\to \infty }{\lim}\int_{\;0}^{\;t}\tau (\tau )\;d\tau <\infty$ by using Barbalat’s lemma [23,24]. Moreover, $q_{1}$ and $q_{2}$ will converge to zero as t→∞; then $a_{r}$ will converge to $a_{m}$ and $v_{r}$ will converge to ${\dot{z}}_{d}$ as t→∞. In consequence, the stability of the micrometer backstepping control system using an amended recurrent Gottlieb polynomials neural network and AACO with the compensated controller can be guaranteed. Additionally, to improve the discontinuous effect of the compensated controller, a smooth approximation of the sign function for k>0 can be represented by 

(31) $$\mathrm{sgn}(q_{2})\approx \frac{\left(e^{kq_{2}}-e^{-kq_{2}}\right)}{\left(e^{kq_{2}}+e^{-kq_{2}}\right)}$$

A cost function that describes the online training algorithm of the amended recurrent Gottlieb polynomials neural network is defined by [25,26]:

(32) $$w_{1}=q_{2}^{2}/2$$

By exploiting the gradient descent method, the adaptive law of the connective weight is given by

(33) $${\dot{z}}_{kj}^{2}=\eta_{1}\;q_{2}ο\;\underline{\underline{\Delta }}\;-\eta_{1}\;\frac{\partial \;w_{1}}{\partial \;h_{k}^{3}}\frac{\partial \;h_{k}^{3}}{\partial \;z_{kj}^{2}}=-\eta_{1}\frac{\partial \;w_{1}}{\partial \;h_{k}^{3}}h_{j}^{2}$$

The above Jacobian term of the controlled system can be rewritten as $\partial \;w_{1}/\partial \;h_{k}^{3}=-q_{2}$. The recurrent weight $z_{ik}^{1}$ from the Jacobian term of the controlled system is given by

(34) $${\dot{z}}_{ik}^{1}\;=-\eta_{2}\frac{\partial \;w_{1}}{\partial \;z_{ik}^{1}}=-\eta_{2}\frac{\partial \text{​}w_{1}}{\partial \;h_{k}^{3}}\;\frac{\partial \;h_{k}^{3}}{\partial \;h_{j}^{2}}\;\frac{\partial \;h_{j}^{2}}{\partial \;h_{i}^{1}}\;\frac{\partial \;h_{i}^{1}}{\partial \;z_{ik}^{1}}=\;\eta_{2}\;q_{2}z_{kj}^{2}GL_{j}(\cdot )r_{i}^{1}(N)h_{k}^{3}(N-1)$$

To improve convergence, the altered ant colony optimization (AACO) is proposed for adjusting two learning rates to obtain two optimal learning rates of the weights in the amended recurrent Gottlieb polynomials neural network. In the basic ant colony optimization (ACO) algorithm [21,22], the pheromone updated values and the probabilistic choice of solution are two important parameters. In the pheromone updated values, the evaporation rate and the length of the best tour are two important factors. In order to improve the pheromone updated rule, the AACO algorithm is proposed and works as follows. 

First, the probabilistic choice of answer [21,22] is defined by:

(35) $$b(d_{ij}|b^{s})=\frac{{\left(\eta_{ij}\right)}^{\gamma }{\left(\upsilon_{ij}\right)}^{\varsigma }}{[\sum_{d_{ij}\in U(b^{s})}{\left(\eta_{il}\right)}^{\gamma }{\left(\upsilon_{il}\right)}^{\varsigma }]},\;\forall d_{ij}\in U(b^{s})\;$$

where $U(b^{s})$ is the available neighborship that is designated the present fractional answer, $b^{s}$; $\upsilon_{ij}$ is the heuristic magnitude regarding the part $d_{ij}$; $\eta_{ij}$ is the pheromone magnitude regarding the part $d_{ij}$; γ can determine magnitude of the pheromone message that belongs to the real number parameter with greater than zero. ς can determine the magnitude of the heuristic message that belongs to a real number parameter greater than zero. The ants put in the answer regarding part $d_{ij}$ to their fractional answer $b^{s}$ by shifting from zenith *i* to zenith *j,* then the ants could attain their ending zenith and finish their entrant answers. The pheromones are preliminarily equal to all zeniths, and design a small magnitude greater than zero. In each tentative, all ants establish their answers until they have either attained the target situation, or the trial outrides some pre-determined limits. Secondly, the renewed rule of the pheromone is as below:

(36) $$\eta_{m,ij}(N+1)=(1-\varphi_{m})\eta_{m,ij}(N)+\varphi_{m}\sum_{k=1}^{M}\Delta \eta_{m}^{}{}_{,\;k,\;best,\;ij},\;m=1,\;2\;\forall d_{ij}\in U(b^{s})\in S_{tr}$$

where $S_{tr}$ is the set of total contender answers originated in the tentative. $\varphi_{m}\in (0,1],\;m=1,\;2\;$ is the evaporation rate in connection with the pheromone magnitude $\eta_{m,ij},\;m=1,\;2$ regarding the learning rate $\eta_{m}(t),\;m=1,\;2$. The magnitude of $\Delta \eta_{m,\;k,\;best,\;ij}^{},\;m=1,\;2$ responds to the number of pheromones in the ant *k* retained on the zeniths that the ant *k* has inquired. Thirdly, the variation magnitude is denoted as below:

(37) $$\Delta \eta_{m}^{}{}_{,\;k,\;best,\;ij}=\frac{1}{\sqrt{[h_{m,k}-(1-c_{m})]c_{m}}}-\frac{1}{\sqrt{[h_{m,\max}^{}-(1-c_{m})]c_{m}}},\;m=1,\;2\;$$

where $h_{m,k},\;m=1,\;2$ is the step count at the ant *k* needed to attain the target situation; $c_{m},\;m=1,\;2$ is the sampling time using seconds; and $h_{m,\max}^{},\;m=1,\;2$ is the maximum value of steps affirmed by a tentative. The magnitude of $(1-c_{m}),\;m=1,\;2$ is available for making the number of pheromones deposit to be closed equal to $1/c_{m},\;m=1,\;2$ when the ant attains the target in exactly one step. The second term in (37) confirms that the pheromones are not renewed when the tentative is ended at the maximum value of time steps and the ant has not yet attained the target. It makes sure that the whole number of pheromones deposited is maximized if all ants search for the shortest route.

In a word, based on two adaptive laws, (33) and (34), for the connective weight adjustment and the recurrent weight adjustment with two optimal learning rates $\eta_{m}^{*}(t),\;m=1,\;2$, the online tuning algorithms of the amended recurrent Gottlieb polynomials neural network are derived. Moreover, the weight estimation errors of the amended recurrent Gottlieb polynomials neural network are fundamentally bounded [27]. The weight estimation errors of the amended recurrent Gottlieb polynomials neural network are bounded, which are required to ensure that the control signal is bounded.

**Remark** **1.** *The key point of the proposed design is to utilize the Lyapunov function for constructing the novel micrometer backstepping control system using an amended recurrent Gottlieb polynomials neural network and AACO with the compensated controller in (23), which reduces the input dimensions of the amended recurrent Gottlieb polynomials neural network controller.*

**Remark** **2.** *The amended recurrent Gottlieb polynomials neural network approximation holds only in a compact set. Thus, the obtained result is semi-global, in the sense that they hold for the compact sets, and there exists a controller with a sufficiently large number of amended recurrent Gottlieb polynomials neural network nodes, such that all the closed-loop signals are bounded.*

## 3. Results

**Figure 5:**
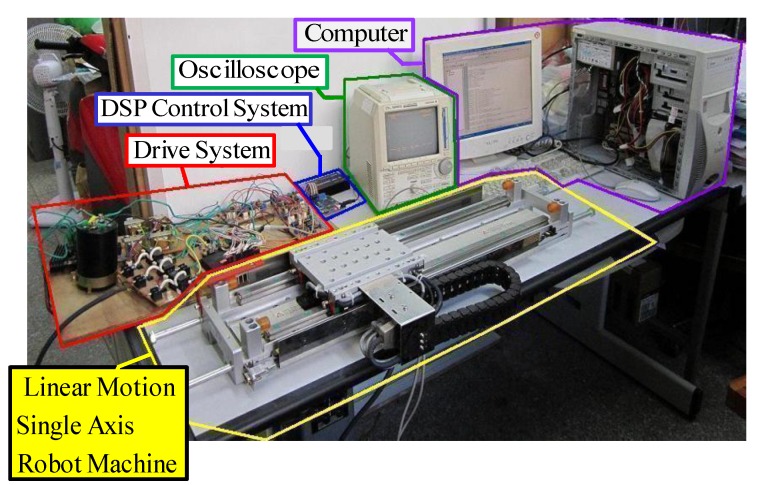
A picture of the experimental set-up of the linear motion single axis robot machine drive system.

The block diagram of the linear motion single axis robot machine drive system by use of the DSP control system is presented in Figure 1. An experimental set-up picture of the linear motion single axis robot machine drive system is shown in Figure 5. 

**Figure 6:**
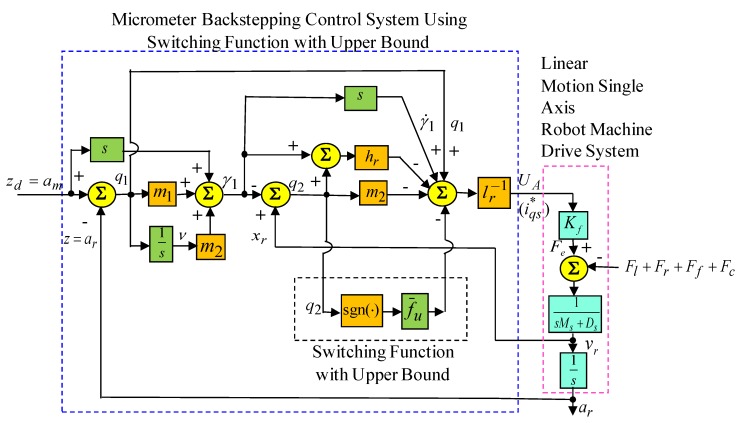
Micrometer backstepping control system using switching function with upper bound.

**Figure 7:**
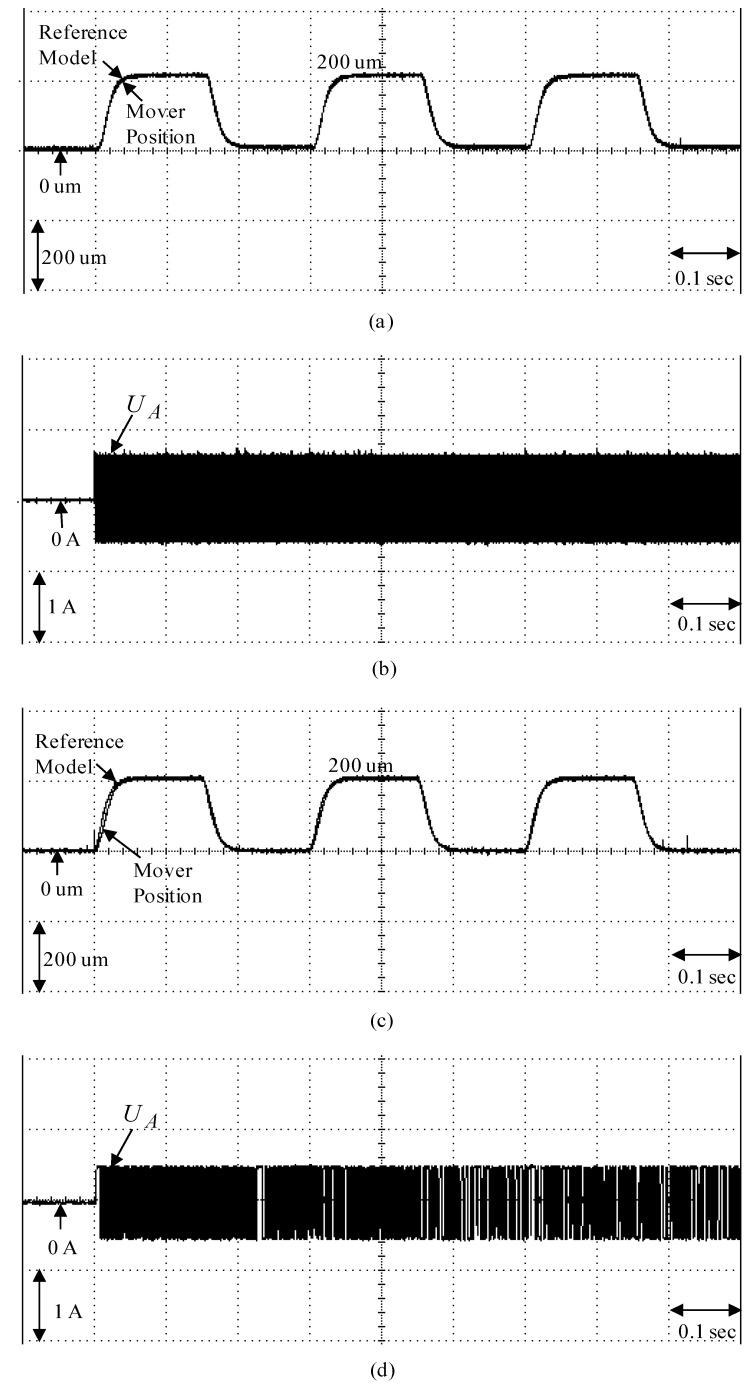
Experimental results of the micrometer backstepping control system using the switching function with an upper bound for the periodic step command: (**a**) mover position in the rated case; (**b**) control intensity in the rated case; (**c**) mover position in the parametric variation case; (**d**) control intensity in the parametric variation case.

**Figure 8:**
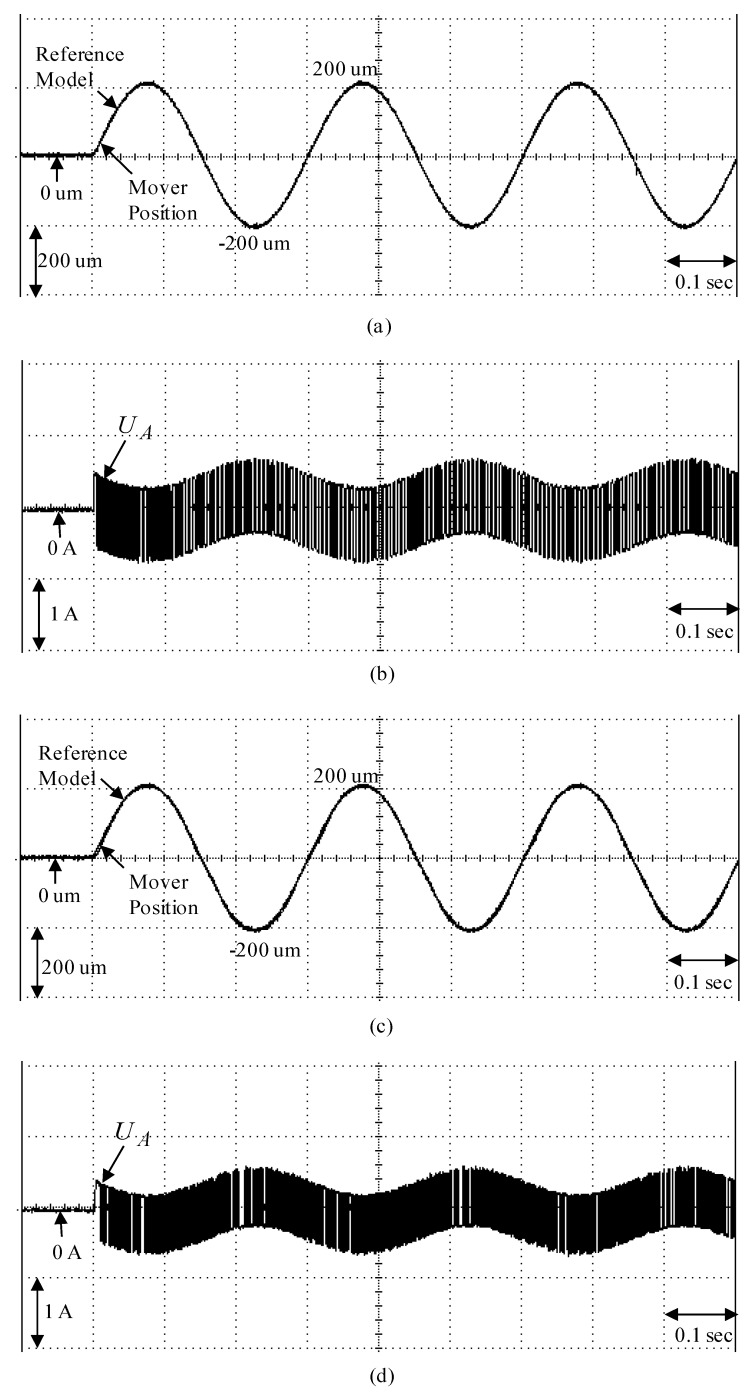
Experimental results of the micrometer backstepping control system using the switching function with an upper bound for the periodic sinusoid command: (**a**) mover position in the rated case; (**b**) control intensity in the rated case; (**c**) mover position in the parametric variation case; (**d**) control intensity in the parametric variation case.

To demonstrate the control performance of the proposed control systems, two cases are provided in the experimentation here. One is the rated case that does not add any load weight onto the mover, and the other is the parametric variation case, which adds the load weight with a 6.3 kg iron disk onto the mover (i.e., it adds to the mover mass with about three times the rated case). The control objective was to drive the mover to move 200 µm, periodically. The experimental results by means of the micrometer backstepping control system using a switching function with an upper bound, which is shown in Figure 6, under the periodic step command and the sinusoidal command in the rated case and the parametric variation case are shown in Figure 7 and Figure 8, respectively. The motion responses of the mover in the rated and parametric variation cases are shown in Figure 7a,c, and Figure 8a,c; the associated control intensities are shown in Figure 7b,d and Figure 8b,d, respectively. Though fine0tracking responses can be obtained by means of the micrometer backstepping control system using the switching function with an upper bound, the oscillation in the control intensity of the linear motion single axis robot machine drive system are bigger due to a large control gain and upper bound.

**Figure 9:**
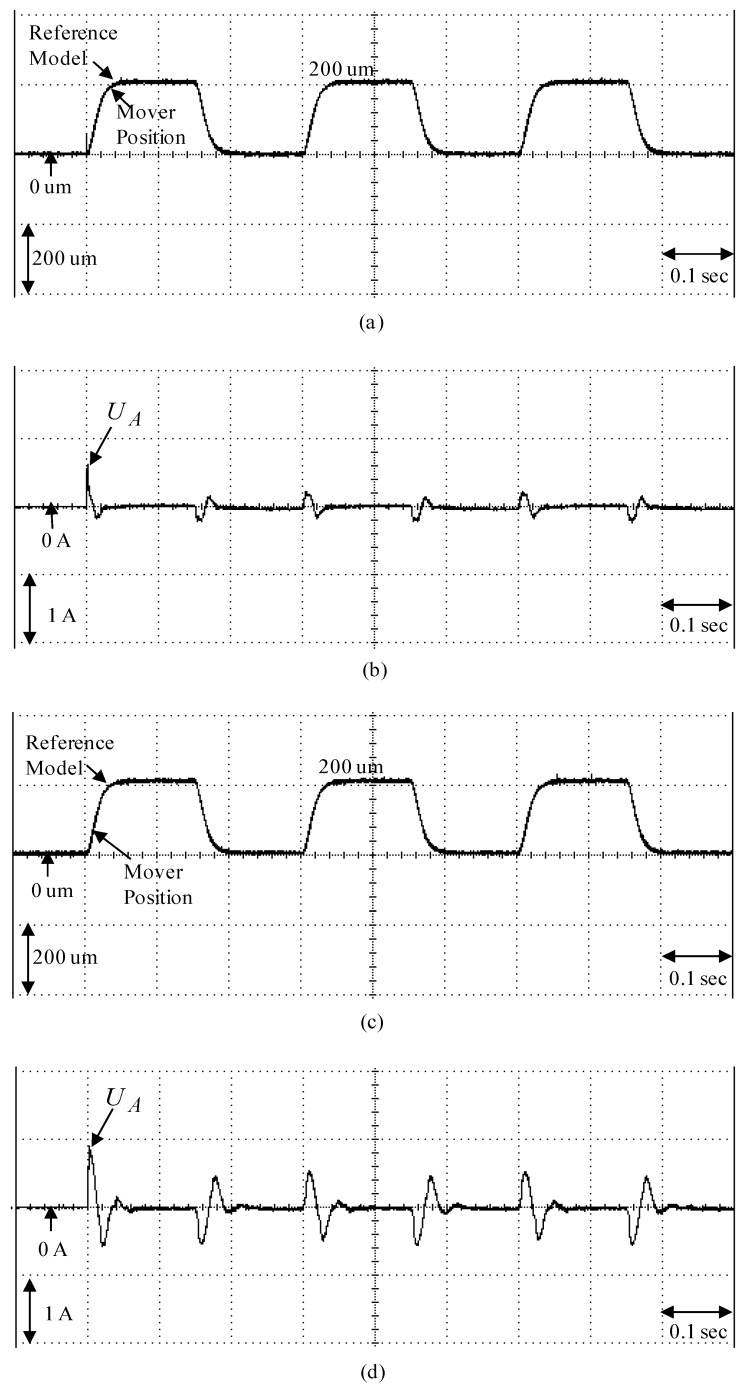
Experimental results of the micrometer backstepping control system using an amended recurrent Gottlieb polynomials neural network and altered ant colony optimization (AACO) with the compensated controller for the periodic step command: (**a**) mover position in the rated case; (**b**) control intensity in the rated case; (**c**) mover position in the parametric variation case; (**d**) control intensity in the parametric variation case.

**Figure 10:**
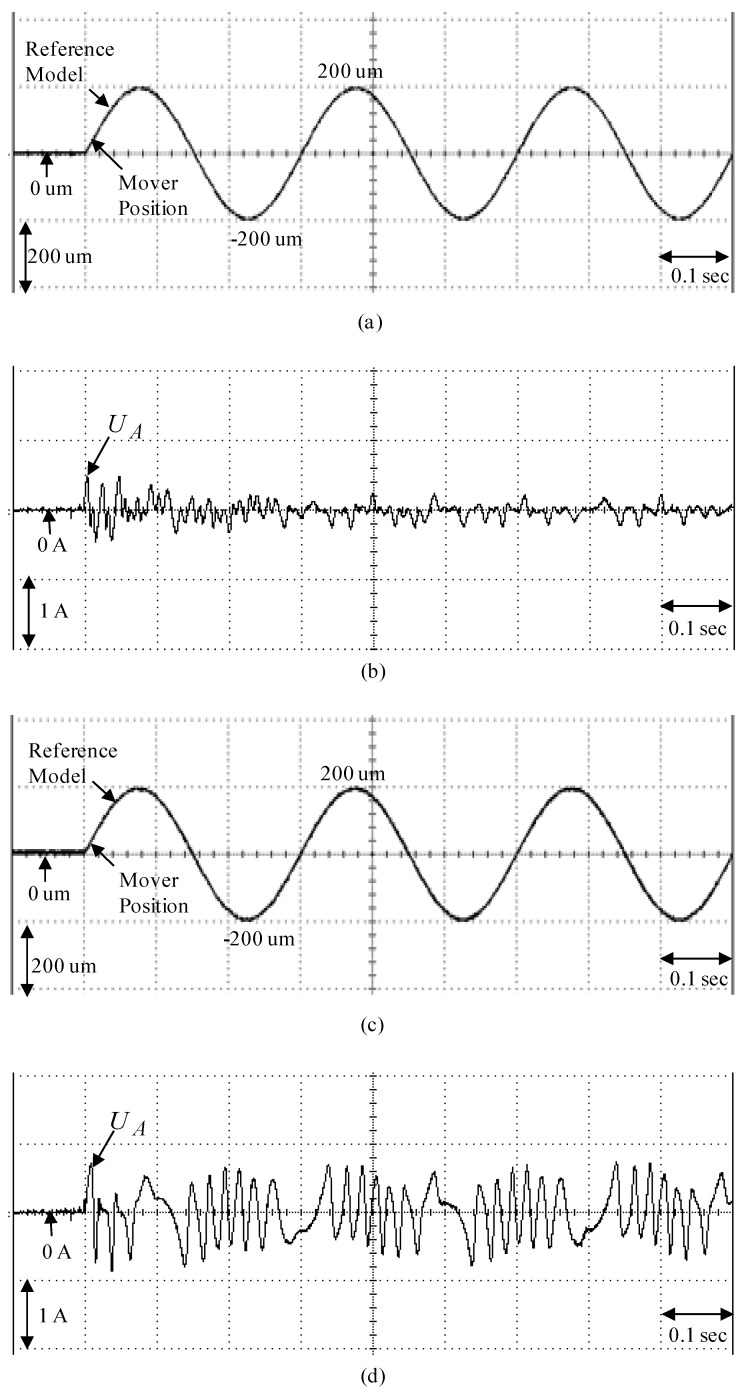
Experimental results of the micrometer backstepping control system using an amended recurrent Gottlieb polynomials neural network and AACO with the compensated controller for the periodic sinusoid command: (**a**) mover position in the rated case; (**b**) control intensity in the rated case; (**c**) mover position in the parametric variation case; (**d**) control intensity in the parametric variation case.

The parameters of the micrometer backstepping control system using an amended recurrent Gottlieb polynomials neural network and AACO with the compensated controller are given as $m_{1}=9$, $m_{2}=4$, ρ=0.1, χ=0.2. The sampling interval of the control processing in the experimentation was set at 1 msec. Furthermore, to show the effectiveness of the control system with a small number of neurons, the used amended recurrent Gottlieb polynomials neural network had 2, 4, and 1 neurons in the input layer, the hidden layer, and the output layer, respectively. The parameter adjustment process remained continually active for the duration of the experimentation. The experimental results of the micrometer backstepping control system using an amended recurrent Gottlieb polynomials neural network and AACO with the compensated controller under the periodic step command and the periodic sinusoidal command in the rated case and the parametric variation case are shown in Figure 9 and Figure 10. The position responses of the mover in the rated case and the parametric variation case are shown in Figure 9a,c and Figure 10a,c; the associated control intensities are shown in Figure 9b,d and Figure 10b,d. However, the robust control performances of the micrometer backstepping control system using an amended recurrent Gottlieb polynomials neural network and AACO with the compensated controller under the occurrence of parametric variations for two kinds of different trajectories are in evidence due to the online adaptive adjustment of the amended recurrent Gottlieb polynomials neural network. From the experimental results, the control performances of the micrometer backstepping control system using an amended recurrent Gottlieb polynomials neural network and AACO with the compensated controller are fine for the tracking of two periodic commands than the micrometer backstepping control system using the switching function with an upper bound.

**Figure 11:**
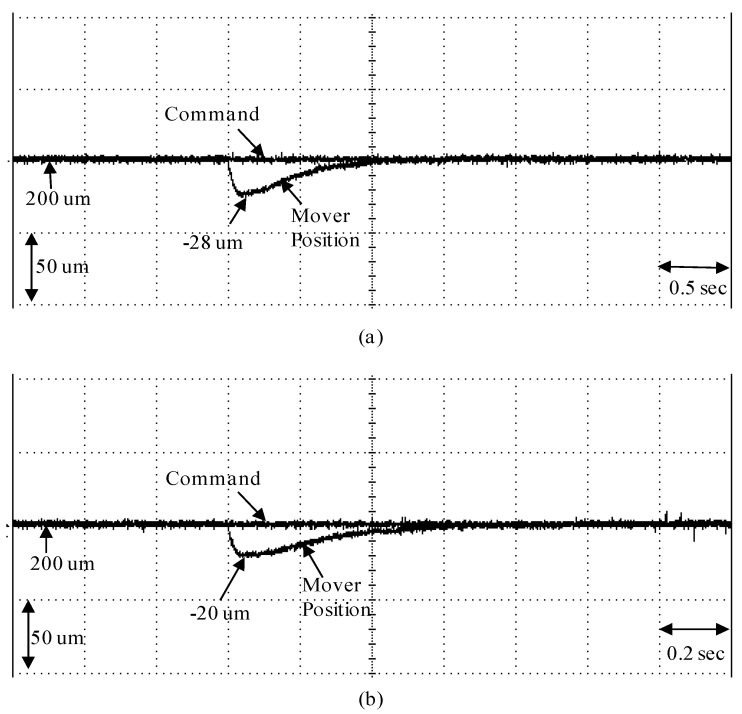
Experimental results of measured mover position response under the step force disturbance with adding load $f_{L}=2\;N$ in the 200um: (**a**) for the micrometer backstepping control system using switching function with upper bound; (**b**) for the micrometer backstepping control system using an amended recurrent Gottlieb polynomials neural network and AACO with the compensated controller.

Finally, the experimental result of the measured mover position response under step force disturbance with adding load $f_{L}=2\;N$ in the 200 µm is shown in Figure 11 in regard to the micrometer backstepping control system using the switching function with upper bound and the micrometer backstepping control system using an amended recurrent Gottlieb polynomials neural network and AACO. Experimental results of measured mover position response for the micrometer backstepping control system using the switching function with an upper bound under step force disturbance with adding load $f_{L}=2\;N$ in the 200 µm is shown in Figure 11a. The experimental result of the measured mover position response for the micrometer backstepping control system using an amended recurrent Gottlieb polynomials neural network and AACO with the compensated controller under step force disturbance with adding load $f_{L}=2\;N$ in the 200 µm is shown in Figure 11b. From these experimental results, the transient response of the micrometer backstepping control system using an amended recurrent Gottlieb polynomials neural network and AACO with the compensated controller is better than the micrometer backstepping control system using the switching function with an upper bound under the load force regulation. However, the robust control performance of the micrometer backstepping control system using an amended recurrent Gottlieb polynomials neural network and AACO with the compensated controller was very outstanding in regard to controlling the linear motion single axis robot machine drive system in the tracking of periodic step and sinusoidal commands under the occurrence of parameter disturbance and the load force regulation, owing to the online adaptive adjustment of the amended recurrent Gottlieb polynomials neural network.

## 4. Discussion

In addition, Table 1 lists some of control performances for the micrometer backstepping control system using a switching function with an upper bound, and the micrometer backstepping control system using an amended recurrent Gottlieb polynomials neural network and AACO with the compensated controller with regard to the experimental results of the five tested cases. 

The maximum errors of $q_{1}$ under the periodic step command in the rated case for the micrometer backstepping control system using a switching function with an upper bound and the micrometer backstepping control system using an amended recurrent Gottlieb polynomials neural network and AACO with the compensated controller are 12 µm and 10 µm, respectively. 

The root-mean-square (RMS) errors of $q_{1}$ under the periodic step command in the rated case for the micrometer backstepping control system using the switching function with an upper bound and the micrometer backstepping control system using an amended recurrent Gottlieb polynomials neural network and AACO with the compensated controller are 8 µm and 6 µm, respectively.

Precision (Relative standard deviation of $q_{1}$) at 200 µm position under the periodic step command in the rated case for the micrometer backstepping control system using the switching function with an upper bound and the micrometer backstepping control system using an amended recurrent Gottlieb polynomials neural network and AACO with the compensated controller are 198.1 µm (±1.01%) and 198.8 µm (±0.91%), respectively. 

Accuracy (Relative error of $q_{1}$) at 200 µm position under the periodic step command in the rated case for the micrometer backstepping control system using the switching function with an upper bound and the micrometer backstepping control system using an amended recurrent Gottlieb polynomials neural network and AACO with the compensated controller are 96.0% (±4.0%) and 97.0% (±3.0%), respectively. 

The maximum errors of $q_{1}$ under the periodic step command in the parametric variation case for the micrometer backstepping control system using a switching function with an upper bound and the micrometer backstepping control system using an amended recurrent Gottlieb polynomials neural network and AACO with the compensated controller are 16 µm and 13 µm, respectively. 

The RMS errors of $q_{1}$ under the periodic step command in the parametric variation case for the micrometer backstepping control system using the switching function with an upper bound and the micrometer backstepping control system using an amended recurrent Gottlieb polynomials neural network and AACO with the compensated controller are 11 µm and 8 µm, respectively. 

Precision (Relative standard deviation of $q_{1}$) at 200 µm position under the periodic step command in the parametric variation case for the micrometer backstepping control system using the switching function with an upper bound and the micrometer backstepping control system using an amended recurrent Gottlieb polynomials neural network and AACO with the compensated controller are 197.6 µm (±1.57%) and 197.9 µm (±1.51%), respectively. 

Accuracy (Relative error of $q_{1}$) at 200 um position under the periodic step command in the parametric variation case for the micrometer backstepping control system using the switching function with an upper bound and the micrometer backstepping control system using an amended recurrent Gottlieb polynomials neural network and AACO with the compensated controller are 94.5% (±5.5%) and 96.0% (±4.0%), respectively. 

The maximum errors of $q_{1}$ under the periodic sinusoid command in the rated case for the micrometer backstepping control system using the switching function with an upper bound and the micrometer backstepping control system using an amended recurrent Gottlieb polynomials neural network and AACO with the compensated controller are 10 µm and 8 µm, respectively. 

The RMS errors of $q_{1}$ under the periodic sinusoid command in the rated case for the micrometer backstepping control system using the switching function with an upper bound and the micrometer backstepping control system using an amended recurrent Gottlieb polynomials neural network and AACO with the compensated controller are 7 µm and 5 µm, respectively. 

Precision (Relative standard deviation of $q_{1}$) at 200 µm position under the periodic sinusoid command in the rated case for the micrometer backstepping control system using the switching function with an upper bound and the micrometer backstepping control system using an amended recurrent Gottlieb polynomials neural network and AACO with the compensated controller are 198.6 µm (±1.00%) and 199.1 µm (±0.90%), respectively. 

Accuracy (Relative error of $q_{1}$) at 200 µm position under the periodic sinusoid command in the rated case for the micrometer backstepping control system using the switching function with an upper bound and the micrometer backstepping control system using an amended recurrent Gottlieb polynomials neural network and AACO with the compensated controller are 96.5% (±3.5%) and 97.5% (±2.5%), respectively. 

The maximum errors of $q_{1}$ under the periodic sinusoid command in the parametric variation case for the micrometer backstepping control system using the switching function with an upper bound and the micrometer backstepping control system using an amended recurrent Gottlieb polynomials neural network and AACO with the compensated controller are 15 µm and 12 µm, respectively. 

The RMS errors of $q_{1}$ under the periodic sinusoid command in the parametric variation case for the micrometer backstepping control system using the switching function with an upper bound and the micrometer backstepping control system using an amended recurrent Gottlieb polynomials neural network and AACO with the compensated controller are 10 µm and 7 µm, respectively. 

Precision (Relative standard deviation of $q_{1}$) at 200 µm position under the periodic sinusoid command in the parametric variation case for the micrometer backstepping control system using the switching function with an upper bound and the micrometer backstepping control system using an amended recurrent Gottlieb polynomials neural network and AACO with the compensated controller are 197.8 µm (±1.47%) and 198.0 µm (±1.40%), respectively. 

Accuracy (Relative error of $q_{1}$) at 200 µm position under the periodic sinusoid command in the parametric variation case for the micrometer backstepping control system using the switching function with an upper bound and the micrometer backstepping control system using an amended recurrent Gottlieb polynomials neural network and AACO with the compensated controller are 95.0% (±5.0%) and 96.5% (±3.5%), respectively. 

The maximum errors of $q_{1}$ under the step force disturbance with adding load $f_{L}=2\;N$ in the 200 µm case for the micrometer backstepping control system using the switching function with an upper bound and the micrometer backstepping control system using an amended recurrent Gottlieb polynomials neural network and AACO with the compensated controller are 28 µm and 20 µm, respectively. 

The RMS errors of $q_{1}$ under the step force disturbance with adding load $f_{L}=2\;N$ in the 200 µm case for the micrometer backstepping control system using the switching function with an upper bound and the micrometer backstepping control system using an amended recurrent Gottlieb polynomials neural network and AACO with the compensated controller are 17 µm and 11 µm, respectively.

Precision (Relative standard deviation of $q_{1}$) at 200 µm position under the step force disturbance with adding load $f_{L}=2\;N$ in the 200 µm case for the micrometer backstepping control system using the switching function with an upper bound and the micrometer backstepping control system using an amended recurrent Gottlieb polynomials neural network and AACO with the compensated controller are 196.5 µm (±2.09%) and 197.1 µm (±2.02%), respectively. 

Accuracy (Relative error of $q_{1}$) at 200 µm position under the step force disturbance with adding load $f_{L}=2\;N$ in the 200 µm case for the micrometer backstepping control system using the switching function with an upper bound and the micrometer backstepping control system using an amended recurrent Gottlieb polynomials neural network and AACO with the compensated controller are 91.5% (±8.5%) and 95.5% (±4.5%), respectively. 

As a result of the micrometer backstepping control system using an amended recurrent Gottlieb polynomials neural network and AACO with the compensated controller has smaller tracking error in comparison with the micrometer backstepping control system using the switching function with an upper bound from Table 1. According to the tabulated measurements, the micrometer backstepping control system using an amended recurrent Gottlieb polynomials neural network and AACO indeed yields better control performance. 

Besides, Table 2 enumerates the feature performance comparisons of the micrometer backstepping control system using the switching function with an upper bound and the micrometer backstepping control system using an amended recurrent Gottlieb polynomials neural network and AACO with the compensated controller in some experimental results. 

Oscillation in the control intensity of the linear motion single axis robot machine drive system for the micrometer backstepping control system using the switching function with an upper bound and the micrometer backstepping control system using an amended recurrent Gottlieb polynomials neural network and AACO with the compensated controller are larger when within 20 µm and smaller within 2 µm, respectively.

The dynamic response of the motion position of the linear motion single axis robot machine for the micrometer backstepping control system using the switching function with an upper bound and the micrometer backstepping control system using an amended recurrent Gottlieb polynomials neural network and AACO with the compensated controller are faster within 0.01 s and fastest within 0.005 s, respectively.

Load regulation capability of the linear motion single axis robot machine for the micrometer backstepping control system using the switching function with an upper bound and the micrometer backstepping control system using an amended recurrent Gottlieb polynomials neural network and AACO with the compensated controller are good (maximum error as 28 µm with adding load in the 200 µm) and best (maximum error as 20 µm with adding load in 200 µm), respectively.

Convergent speed of the motion position of the linear motion single axis robot machine for the micrometer backstepping control system using the switching function with an upper bound and the micrometer backstepping control system using an amended recurrent Gottlieb polynomials neural network and AACO with the compensated controller are faster within 0.002 s and fastest within 0.001 s, respectively.

The position tracking error of the motion position of the linear motion single axis robot machine for the micrometer backstepping control system using the switching function with an upper bound and the micrometer backstepping control system using an amended recurrent Gottlieb polynomials neural network and AACO with the compensated controller are medium with maximum error of $q_{1}$ from 10 µm to 16 µm and small with a maximum error of $q_{1}$ from 8 µm to 13 µm, respectively.

The rejection capability for parameters’ disturbance of the motion position of the linear motion single axis robot machine for the micrometer backstepping control system using the switching function with an upper bound and the micrometer backstepping control system using an amended recurrent Gottlieb polynomials neural network and AACO with the compensated controller are good with maximum error of $q_{1}$ within 16 µm and better with a maximum error of $q_{1}$ within 13 µm, respectively.

The learning rate of the amended recurrent Gottlieb polynomials neural network for the micrometer backstepping control system using the switching function with an upper bound and the micrometer backstepping control system using an amended recurrent Gottlieb polynomials neural network and AACO with the compensated controller are none and variable (optimal rate), respectively.

The various performances in Table 2 for the micrometer backstepping control system using an amended recurrent Gottlieb polynomials neural network and AACO with the compensated controller are better than the micrometer backstepping control system using the switching function with an upper bound with regard to the oscillation in the control intensity, the dynamic response, the load regulation capability, the convergent speed, the position tracking error, and the rejection capability of parameter disturbance. 

## 5. Conclusions

In this paper, the micrometer backstepping control system using the amended recurrent Gottlieb polynomials neural network and AACO with the compensated controller was proposed to control the linear motion single axis robot machine drive system under the occurrence of parameter disturbance for the position tracking of periodic reference inputs. The important contributions of this paper are as follows: (1) The DSP-based current-regulation PWM control scheme has been successfully applied to control the linear motion single axis robot machine drive system; (2) the micrometer backstepping control system using an amended recurrent Gottlieb polynomials neural network with the compensated controller has been successfully derived according to the Lyapunov function for diminishing the lumped uncertainty effect; (3) to achieve high-precision control performance, an adaptive law of the amended recurrent Gottlieb polynomials neural network based on the Lyapunov function has been successfully applied for estimating the lumped uncertainty; (4) an error-estimated law of the compensated controller has been successfully used to compensate the estimated error; and (5) the AACO has been successfully used for regulating two variable learning rates in the weights of the amended recurrent Gottlieb polynomials neural network to speed up the convergent speed. The various performances verified by the experimental results in Table 1 and Table 2 for the micrometer backstepping control system using an amended recurrent Gottlieb polynomials neural network and AACO with the compensated controller are better than the micrometer backstepping control system using a switching function with an upper bound.

## Figures and Tables

**Table 1:** Performance comparison of control systems.

	Control system and five tested cases	micrometer backstepping control system using switching function with upper bound				
Performance		under the periodic step command in the rated case	under the periodic step command in the parametric variation case	under the periodic sinusoid command in the rated case	under the periodic sinusoid command in the parametric variation case	under the step force disturbance with adding load $f_{L}=2\;N$ in the 200 µm case
Maximum error of $q_{1}$		12 µm	16 µm	10 µm	15 µm	28 µm
RMS error of $q_{1}$		8 µm	11 µm	7 µm	10 µm	17 µm
Precision (Relative standard deviation of $q_{1}$) at 200 µm position		198.1 µm (±1.01%)	197.6 µm (±1.57%)	198.6 µm (±1.00%)	197.8 µm (±1.47%)	196.5 µm (±2.09%)
Accuracy (Relative error of $q_{1}$) at 200 µm position		96.0% (±4.0%)	94.5% (±5.5%)	96.5% (±3.5%)	95.0% (±5.0%)	91.5% (±8.5%)
	Control system and five tested cases	micrometer backstepping control system by using an amended recurrent Gottlieb polynomials neural network and AACO with the compensated controller				
performance		under periodic step command in the rated case	under the periodic step command in the parametric variation case	under the periodic sinusoid command in the rated case	under the periodic sinusoid command in the parametric variation case	under the step force disturbance with adding load $f_{L}=2\;N$ in the 200um case
Maximum error of $q_{1}$		10 µm	13 µm	8 µm	12 µm	20 µm
RMS error of $q_{1}$		6 µm	8 µm	5 µm	7 µm	9 µm
Precision (Relative standard deviation of $q_{1}$) at 200 µm position		198.8 µm (±0.91%)	197.9 µm (±1.51%)	199.1 µm (±0.90%)	198.0 µm (±1.40%)	197.1 µm (±2.02%)
Accuracy (Relative error of $q_{1}$) at 200 µm position		97.0% (±3.0%)	96.0% (±4.0%)	97.5% (±2.5%)	96.5% (±3.5%)	95.5% (±4.5%)

**Table 2:** Feature performance comparisons of control systems.

	Control system	micrometer backstepping control system using switching function with upper bound	micrometer backstepping control system using an amended recurrent Gottlieb polynomials neural network and AACO with the compensated controller
Feature Performance			
Oscillation in the control intensity of the linear motion single axis robot machine drive system		Larger within 20 µm	Smaller within 2 µm
Dynamic response of the motion position of the linear motion single axis robot machine		Faster within 0.01 s	Fastest within 0.005 s
Load regulation capability of the linear motion single axis robot machine		Good (maximum error as 28 µm with adding load in the 200 µm)	Best (maximum error as 20 µm with adding load in the 200 µm)
Convergent speed of the motion position of the linear motion single axis robot machine		Faster within 0.002 s	Fastest within 0.001 s
Position tracking error of the motion position of the linear motion single axis robot machine		Middle with maximum error of $q_{1}$ from 10 µm to 16 µm	Small with maximum error of $q_{1}$ from 8um to 13 µm
Rejection capability for parameters disturbance of the motion position of the linear motion single axis robot machine		Good with maximum error of $q_{1}$ within 16um	Better with maximum error of $q_{1}$ within 13 µm
Learning rate of the amended recurrent Gottlieb polynomials neural network		None	Vary (optimal rate)
